# A fond farewell

**DOI:** 10.1120/jacmp.v13i6.4237

**Published:** 2012-11-08

In 2007, I was invited to serve as the Editor‐in‐Chief of the *Journal of Applied Clinical Medical Physics*, and in 2008, with the publication of Volume 9, I began my term. I accepted the position with a personal goal of serving for five years. With the conclusion of Volume 13, I have now served five years, so the time has come for me to step down and provide the *JACMP* with an opportunity for fresh leadership.

Anticipating the end of my term, over a year ago I notified the American College of Medical Physics, the organization that sponsored the *JACMP*, that I would be stepping down at the conclusion of 2012, with the intention of staying on for at least a year after management of the *JACMP* was taken over by the American Association of Physicists in Medicine (AAPM), to effect a smooth transition. After a nationwide search for a new Editor‐in‐Chief, the AAPM recently appointed Dr. Michael Mills to replace me. Many of you remember Dr. Mills as the previous Editor‐in‐Chief of the *JACMP*.

The past five years have been an exciting time for the Journal and for me as Editor‐in‐Chief. The first accomplishment that occurred during this time period was the expansion of the scope of the *JACMP* into an international journal. We have added two key organizations as sponsors: the Canadian Organization of Medical Physicists and the International Organization of Medical Physics, along with the addition of several Associate Editors nominated by these two organizations. Consequently, we now have a much broader reader base, as well as a much broader author base than we had previously. A second accomplishment has occurred in conjunction with the AAPM in that many *JACMP* articles are now available online on the AAPM website, so that medical physicists can obtain continuing education credits. Finally, we have experienced major growth in the *JACMP* with not only a greater number of submissions during the past five years (Table 1), but also a greater number of manuscripts published, as well (Table 2). As a consequence of the increased number of published manuscripts, with Volume 13 we initiated publishing six issues per year instead of four issues per year.

**Table 1:** Number of JACMP submissions (2008–2012^a^)

*Year* ‐ *Quarter*	# *of Submissions*
2008 – q1	33
2008 – q2	44
2008 – q3	32
2008 – q4	33
2009 – q1	55
2009 – q2	44
2009 – q3	46
2009 – q4	45
2010 – q1	53
2010 – q2	69
2010 – q3	52
2010 – q4	61
2011 – q1	71
2011 – q2	49
2011 – q3	64
2011 – q4	73
2012 – q1	87
2012 – q2	79
2012 – q3	92

^a^As at the end of the 3rd quarter 2012.

**Table 2:** Number of JACMP published manuscripts (2008–2012).

*Year*	*Volume*	*Number*	*# Manuscripts*	*Volume Total*
2008	9	1	9	
		2	11	
		3	17	
		4	19	56
2009	10	1	15
		2	15	
		3	19	
		4	25	74
2010	11	1	27
		2	19	
		3	23	
		4	29	98
2011	12	1	26	
		2	34	
		3	24	
		4	27	111
2012	13	1	21
		2	19	
		3	16	
		4	19	
		5	29	
		6	31	135

Finally, I would like to acknowledge the difficult and time‐consuming work of our Associate Editors and reviewers in maintaining the high quality of manuscripts published in the *JACMP*. The names of Associate Editors can be found below, and the names of reviewers can be found in a Supplementary File.

Associate Editors (including Guest Associate Editors) for Volume 13 include the following: Nzhde Agazaryan, Salahuddin Ahmad, John Antolak, Pat Cadman, John Bayouth, Charles Bloch, Pat Cadman, Marco Carlone, Nathan Childress, Laurence Court, Luca Cozzi, Mini Das, Larry DeWerd, Bill Erwin, Vladimir Feygelmann, Kent Gifford, Ed Jackson, Jennifer Johnson, Tommy Knöös, Stephen Kry, Rajat Kudchadker, Harish Malhotra, Pietro Mancosu, Osama Mawlawi, Charles Mayo, Moyed Miften, Mike Mills, Eduardo Moros, Firas Mourtada, Ben Nelms, Jennifer O'Daniel, Niko Papanikolaou, Donald Peck, Matt Podgorsak, Joann Prisciandaro, Jim Rodgers, John Rong, Yi Rong, Isaac Rosen, Surendra Rustgi, Narayan Sahoo, Bill Salter, Mehrdad Sarfaraz, Jeff Shepard, Chengyu Shi, Alf Siochi, Eric Slessinger, Jason Stafford, Lu Wang, Chuck Willis, Kamil Yenice, Geoffrey Zhang, and Ronald Zhu.

Thank you to our Associate Editors and our reviewers, and especially to our authors, without whose contributions we would have no journal. My best wishes go to my successor, Dr Mills.

George Starkschall, PhD

Editor‐in‐Chief

## Supporting information

Supplementary Material Files

Supplementary Material Files

