# Supplementary material for: A fond farewell
Source: J Appl Clin Med Phys. 2012 Nov 8;13(6):1–2. doi: 10.1120/jacmp.v13i6.4237 (PMC5718551; doi:10.1120/jacmp.v13i6.4237)
Supplement: Supplementary file 1 — Supplementary Material Files [file ACM2-13-001-s001.pdf]

2012 Reviewer Record

(10/1/2011-9/30/2012)

Adil	Akhtar
Nabil	Adnani
Munir	Ahmad
Nasser	Ahmadi
Ergun	Ahunbay
Parham	Alaei
Hania	Al-Hallaq
Imad	Ali
Paola	Alvarez
Richard	Amos
Christopher	Anker
Louis	Archambault
Maria	Asparadakis
Ebenezer	Babu
Daniel	Bailey
Mohammed	Bakhtiari
James	Balter
Peter	Balter
Ande	Bao
Laura	Bartol
Luc	Beaulieu
James	Bedford
Gloria	Beyer
Ravi	Bhatnagar
Tewfik	Bichay
Peter	Biggs
Charles	Bloch
Kirsten	Boedeker
Alessandra	Bolsi
Elisabeth	Bossart
Hugo	Bouchard
Samuel	Brady
Edward	Brandner
Tina	Briere
Kristy	Brock
Wayne	Butler
Jing	Cai
Minsong	Cao
Mauro	Carrara
Richard	Castillo
George	Cernica
Chee-Wai	Chang
Zheng	Chang

Yong	Chen
Yu	Chen
Joel	Cheung
Nathan	Childress
Jongmin	Cho
James	Chow
Michael	Christophanus
Heeteak	Chung
Brenda	Clark
Peter	Colley
Laurence	Court
Luca	Cozzi
Tim	Craig
Richard	Crilly
J Adam	Cunha
Bruce	Curran
Joanna	Cygler
Prajnan	Das
Melanie	Davidson
Meisong	Ding
Xuanfeng	Ding
Blake	Dirksen
Laura	Drever
Weiliang	Du
Jun	Duan
David	Eaton
Issam	el Naqa
Eric	Elder
William	Erwin
Alessandro	Facure
Jessica	Fagerstrom
Pascal	Fenoglietto
Vladimir	Feygelman
Luis	Fong de los Santos
Kenneth	Forster
Weihua	Fu
Regina	Fulkerson
Isabelle	Gagnon
Jose	Garcia
Olivier	Gayou
William	Geiser
Lee	Gerig
Jacob	Gersh
Marco	Gianelli
Kent	Gifford
Michael	Gillin
Martin	Glegg

Murty	Goddu
Devon	Godfrey
Steven	Goetsch
Krzysztof	Gorney
Domingo	Granero
Ryan	Grant
Edward	Graves
Travis	Greene
Jimm	Grimm
Sebastian	Gros
Bingqi	Guo
Madhup	Gupta
Alonso	Gutierrez
Scott	Hadley
Uli	Haedlinger
Joe	Hanley
William	Harms
Alicia	Harris
Mathieu	Hatt
John	Hazle
Tania	Herman
Patrick	Higgins
Emily	Hirata
Linda	Hong
Murshed	Hossain
Alan	Hounsell
Rebecca	Howell
Wen	Hsi
Chi-Wen	Hsieh
Annie	Hsu
Chia-Ho	Hua
Long	Huang
Semra	Icer
Ali	Imad
Mohammed	Islam
Shirish	Jani
Hazim	Jaradat
Juergen	Jenne
Andrew	Jensen
Hosang	Jin
Daniel	Johnson
Bernard	Jones
Jimmy	Jones
James	Jordan
Chandra	Joshi
Paul	Jurisinic
Cheenu	Kappadath

Pantelis	Karaiskos
Paul	Keall
Robin	Kelly
James	Kerns
Kevin	Khadivi
Leonard	Kim
Yongbok	Kim
Yusing	Kim
Rajesh	Kinhikar
Steven	Kirsner
Kelly	Kisling
Michael	Kissick
Alex	Krafft
Gebrielle	Kragl
Sergei	Kriminski
Amanda	Krintz
Thomas	Kron
Stephen	Kry
Lichung	Ku
Rajat	Kudchadker
Narayan	Kulkarni
Rajesh	Kumar
Lalith	Kumaraswamy
Ron	Lalonde
Kwok	Lam
James	Lamb
Stephanie	Lang
Katja	Langen
Ulrich	Langner
Joerg	Lehmann
Fritz	Lerma
Baojun	Li
Guang	Li
Haisen	Li
Xiang	Li
Haibo	Lin
Lan	Lin
Liyong	Lin
Anthony	Liu
Feghong	Liu
Fengzhong	Liu
Wu	Liu
Xinming	Liu
Yaxi	Liu
Ralf	Loeffler
Dershan	Luo
Yulia	Lyatskaya

Jingfei	Ma
Lijun	Ma
Miller	MacPherson
Maria	Mamalui-Hunter
James	Marbach
Rebecca	Marsh
Christopher	Mart
Anne-Catherine	Martinson
Bryan	Mason
Chance	Mathiessen
Jason	Matney
Panayotis	Mavroidis
Charles	Mayo
Malcolm	McEwen
Andrea	McNiven
Sanford	Meeks
Ali	Meigooni
Robert	Meiler
Adam	Melancon
Geetha	Menon
Moyed	Miften
Rafael	Mockli
Andrea	Molineau
Shinichiro	Mori
Vadim	Moskvin
Firas	Mourtada
Shahid	Naqvi
Ganesh	Narayanaswamy
Daryl	Nazareth
Alois	Ndlovu
Ben	Nelms
Giorgia	Nicolini
Thomas	Nishino
Paige	Nitsch
Frederick	Nordstrom
Joe	Och
Jennifer	O'Daniel
Jared	Ohrt
Zoubir	Ouhib
Matthew	Pacella
Niko	Papanikolaou
Lech	Papiez
Sungyong	Park
William	Parker
David	Pearson
Julian	Perks
Paula	Petti

Uwe	Pietrzyk
Sam	Pokharel
Antonio	Popescu
Richard	Popple
Vincenzo	Positano
Anand	Prabhu
Mich	Price
Kiley	Pulliam
Thomas	Purdie
Zhihua	Qi
Michelle	Quan
Javed	Rahimian
Frank	Ranallo
Min	Rao
Premia	Rassiah-Szegedi
Satyapal	Rathee
Laura	Rechner
Chester	Reft
Jay	Reiff
Nicholas	Remmes
Susan	Richardson
Edward	Ring
Mark	Rivard
John	Roeske
Xinjiang	Rong
J	Rottman
Lindsey	Runyan
Ali	Sadeghi-Naini
Greg	Salomons
Ehsan	Samei
Andappa	Sankar
Vikren	Sarkar
Otto	Sauer
Daniel	Scanderbeg
Stefan	Scheib
Eduard	Schreibmann
John	Schreiner
Alireza	Sedaghat
Amarjit	Sen
Anil	Sethi
Bilal	Shanine
Gregory	Sharp
Michael	Sharpe
Jeff	Shepard
Tony	Shepherd
Chengyu	Shi
Almon	Shiu

Jose	Silva
Peter	Situ
Eva	Sjolin
Ron	Sloboda
Benjamin	Smith
Gregory	Smith
Wendy	Smith
Chris	Soares
Emile	Soisson
William	Song
Marc	Sontag
Ingrid	Spadinger
Michael	Speiser
David	Spencer
Emiliano	Spezi
Chris	Stacey
Jason	Stafford
Sotirios	Stathakis
Sotirios	Stathkis
Robin	Stern
Doonna	Stevens
Matthew	Studenski
Zhong	Su
Brinda	Subramanian
Yelin	Suh
Mark	Supanich
Kazumichi	Suzuki
Martin	Szegedi
Shikui	Tang
James	Tanyi
Valery	Taranenko
Brian	Taylor
Steve	Tenn
Maria	Thor
Joseph	Ting
Uwe	Titt
Doron	Todor
Bryan	Tolenaar
Seth	Toner
Giovanni	Tosi
Abdelkader	Toutaoui
Wolfgang	Ullrich
Jacob	Van Dyk
Ann	Van Esch
Monique	van Prooijen
Sastry	Vedam
Michael	Velec

Frank	Verhaegen
Jose	Villareal-Barajas
Maria	Vlachaki
Shada	Wadi-Ramahi
Daniela	Wagner
Brian	Wang
Chuang	Wang
Dongxu	Wang
Jia	Wang
Jihong	Wang
Xiaochun	Wang
Yi	Wang
Yizhen	Wang
Zhongmin	Wang
Rebecca	Weinberg
Richard	Wendt
David	Westerly
Krishni	Wijesooriya
Juergen	Wilbert
Charles	Willis
Twyla	Willoughby
H	Wooten
Binbin	Wu
Richard	Wu
Tianming	Wu
Ping	Xia
Hong	Xiang
Lei	Xing
Li	Xiong
Weijun	Xiong
Guanghua	Yan
Yulong	Yan
Kai	Yang
Ming	Yang
Yingli	Yang
Zhitong	Yang
Ravindra	Yaparpalvi
Shigen	Yokoyama
Sua	Yoo
Jiankui	Yuan
Grace	Zeng
Da	Zhang
Hualin	Zhang
Qinghui	Zhang
Sean	Zhang
Tianyu	Zhao
Dandan	Zheng

Dengsong	Zhu
Xiaowei	Zhu
Yang Ming	Zhu
